# Structure Meets Function: Dissecting Fucoxanthin’s Bioactive Architecture

**DOI:** 10.3390/md23110440

**Published:** 2025-11-15

**Authors:** Patrícia Nogueira, Victória Bombarda-Rocha, Rita Tavares-Henriques, Mariana Carneiro, Emília Sousa, Jorge Gonçalves, Paula Fresco

**Affiliations:** 1 Laboratório de Farmacologia, Departamento de Ciências do Medicamento, Faculdade de Farmácia, Universidade do Porto, Rua de Jorge de Viterbo Ferreira 228, 4050-313 Porto, Portugal; up201704254@edu.ff.up.pt (P.N.); up202200055@edu.ff.up.pt (V.B.-R.); arhenriques@ff.up.pt (R.T.-H.); pfresco@ff.up.pt (P.F.); 2 UCIBIO Applied Molecular Biosciences Unit, Mechanistic Pharmacology and Pharmacotherapy, Associate Laboratory i4HB, Institute for Health and Bioeconomy, Faculty of Pharmacy, University of Porto, 4050-313 Porto, Portugal; 3 Necton S.A., Belamandil s/n, 8700-152 Olhão, Portugal; mariana.carneiro@necton.pt; 4 Laboratório de Química Orgânica e Farmacêutica, Departamento de Ciências Químicas, Faculdade de Farmácia, Universidade do Porto, Rua de Jorge de Viterbo Ferreira 228, 4050-313 Porto, Portugal; esousa@ff.up.pt; 5 CIIMAR—Centro Interdisciplinar de Investigação Marinha e Ambiental, Universidade do Porto, Terminal de Cruzeiros do Porto de Leixões, 4450-208 Matosinhos, Portugal

**Keywords:** fucoxanthin, metabolites, synthetic derivatives, signalling pathways, SARs

## Abstract

Fucoxanthin (Fx), a marine xanthophyll carotenoid, has attracted considerable scientific attention due to its wide-ranging biological activities, including antioxidant, anti-inflammatory, anti-obesity, and anticancer effects. Despite its substantial therapeutic potential, the clinical application of Fx and its derivatives remains constrained by their structural complexity, low chemical stability, and limited bioavailability. This review offers a thorough and up-to-date overview of Fx, encompassing its primary natural sources, the metabolic biotransformation to fucoxanthinol (FxOH) and amarouciaxanthin A—metabolites whose bioactive properties significantly contribute to the observed in vivo effects—and the molecular mechanisms underlying the biological activities of Fx and its metabolites, with emphasis on their modulation of key intracellular signalling pathways involved in inflammation, lipid metabolism, and cell proliferation. Furthermore, it explores how targeted structural modifications may enhance the pharmacokinetic profiles and expand the therapeutic potential of Fx-based compounds, while highlighting promising strategies for their optimisation. By integrating insights from pharmacology, biochemistry, and synthetic chemistry, this work aims to guide future efforts in the rational design of marine-derived bioactive agents and underscores the value of marine biodiversity in therapeutic innovation.

## 1. Introduction

The blue economy promotes the sustainable use of ocean resources to drive economic growth, improve living standards, and enhance ecosystem health [1]. This has highlighted the immense and largely unexplored potential of marine biodiversity. In this context, marine-derived bioactive compounds have emerged as promising candidates for the development of novel therapeutic agents [1]. One such compound is fucoxanthin (Fx), a xanthophyll carotenoid predominantly found in macroalgae (*Phaeophyceae*) and microalgae (*Bacillariophyta* and *Haptophyta*), which has attracted significant scientific interest due to its diverse biological activities, including antioxidant, anti-inflammatory, anti-obesity and anticancer properties [2,3,4,5,6].

Despite growing interest in Fx, the production of its natural or synthetic metabolites and derivatives remains limited. In addition to the low abundance and instability of natural metabolites, the chemical synthesis of Fx derivatives is complicated by the molecule’s structural complexity, particularly the presence of reactive functional groups and conjugated bonds [7]. These difficulties limit the development of more stable, potent and bioavailable analogues [8,9].

It is essential to understand how Fx and its analogues act mechanistically at the cellular level, particularly in signalling pathways such as NF-κB, Nrf2, or PPAR, to justify their therapeutic potential and guide structural optimisation. In this context, structure-activity relationship (SAR) studies play a central role. By establishing which structural modifications influence biological activity, SAR studies allow a deeper understanding of the mode of action of compounds and guide strategies for synthesising new derivatives with improved pharmacological properties. This integrated approach, combining metabolism, biological activity, and modulation of signalling pathways, is vital for advancing the biotechnological utilisation of Fx and its derivatives.

The aim of this review is to provide a comprehensive and up-to-date overview of Fx, addressing its natural origin, metabolic pathways, molecular mechanisms of action, and SAR studies. The ultimate objective of this work is, therefore, twofold: firstly, to deepen our understanding of the therapeutic potential of Fx metabolites and derivatives; and secondly, to highlight the current challenges and outline potential strategies for their future development.

## 2. Fucoxanthin Biological Activities

### 2.1. Natural Sources

Marine organisms are a prolific source of diverse and innovative bioactive molecules. The extraction of bioactive compounds from marine resources exemplifies the vast potential of the blue economy to contribute to the world’s economic development while promoting sustainability [1]. As interest in marine-derived molecules with pharmaceutical, nutraceutical, and industrial applications continues to grow, the exploration of ocean-based resources offers a promising avenue for innovation that aligns with global efforts toward a more sustainable and circular bioeconomy [1].

**Figure 1:**
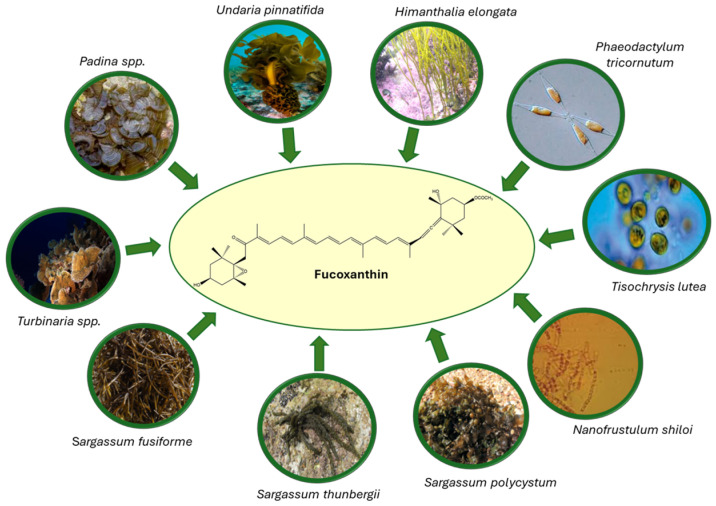
Representative brown macroalgae and microalgae species as natural sources of fucoxanthin (Fx).

Some of the molecules derived from marine organisms exhibit remarkable potential for the development of novel therapeutic agents [10]. Among these bioactive compounds, Fx—an orange-brown xanthophyll carotenoid—stands out for its wide distribution and biological relevance. Fx is naturally abundant in several species of brown macroalgae, including *Undaria pinnatifida* (wakame), *Sargassum fusiforme*, *Sargassum polycystum*, *Sargassum thunbergii*, *Padina* spp., *Turbinaria* spp., and *Himanthalia elongata* [11,12,13,14,15,16,17,18,19], as well as in various microalgae such as diatoms (*Phaeodactylum tricornutum* [20] and *Nanofrustulum shiloi* [21]) and golden-brown microalgae (*Tisochrysis lutea*) [22]. Owing to its distinctive chemical structure and diverse biological properties, Fx has recently attracted growing scientific interest for its antioxidant, anti-inflammatory, anticancer, and anti-obesity potential (Figure 1).

### 2.2. Biosynthetic Pathways

In view of Fx’s putative therapeutic potential, there is an increasing need to understand the biosynthetic origin of Fx. Recent studies using the model diatom *Phaeodactylum tricornutum* have clarified key enzymatic steps involved in Fx formation, paving the way for sustainable production strategies [23,24].

**Figure 2:**
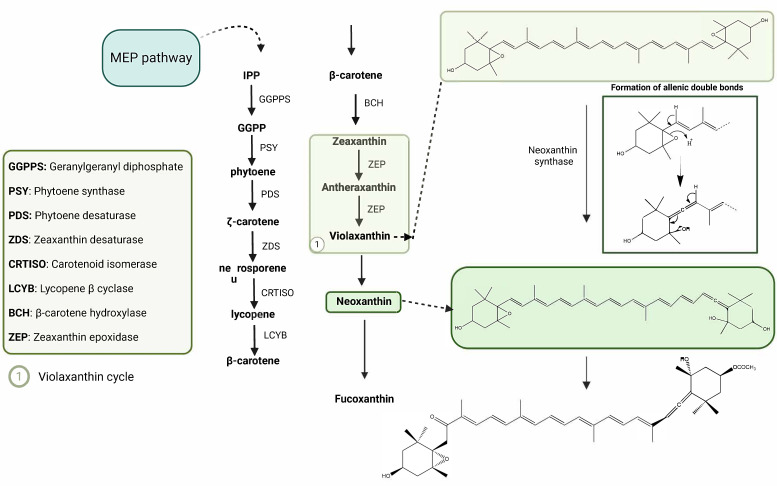
Proposed biosynthetic pathway of fucoxanthin (Fx) in *Phaeodactylum tricornutum*, illustrating its progression from β-carotene via the violaxanthin cycle and key enzymatic steps. MEP pathway: methylerythritol phosphate pathway. Chemical structures were created using ChemDraw Ultra 12.0. Created in BioRender. Nogueira, P. (2025) https://BioRender.com/rs7t99m (accessed on 13 June 2025).

As Figure 2 illustrates, Fx biosynthesis is initiated by the production of isopentenyl pyrophosphate (IPP) via the mevalonate (MVA) or methylerythritol phosphate (MEP) pathways [24,25]. IPP is converted by geranyl pyrophosphate synthase (GGPS) into geranylgeranyl diphosphate (GGPP), which is then used by phytoene synthase (PSY) to produce phytoene, the first intermediate in the production of carotenoids [24,25] which is then converted into lycopene.

**Figure 3:**
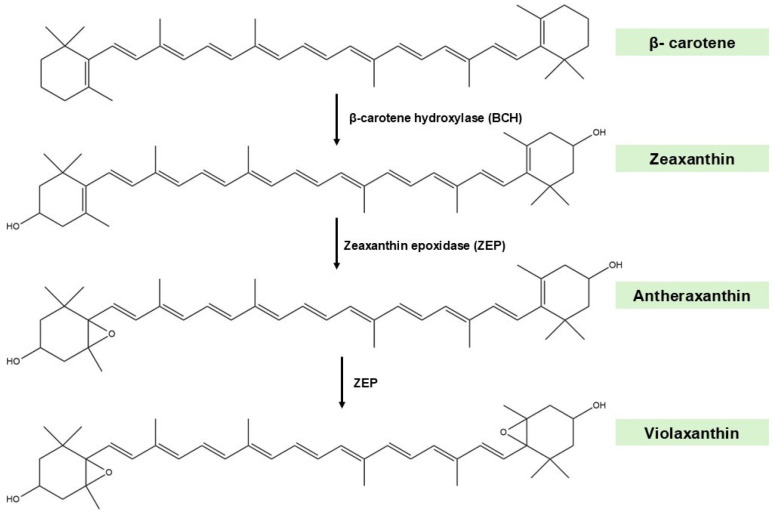
Initial phase of the fucoxanthin (Fx) biosynthetic pathway. The diagram illustrates the enzymatic conversion of β-carotene into violaxanthin via zeaxanthin and antheraxanthin, catalysed by β-carotene hydroxylase (BCH) and zeaxanthin epoxidase (ZEP). Chemical structures were created using ChemDraw Ultra 12.0.

It has been established that the conversion of phytoene to lycopene occurs through a sequence of desaturation and isomerisation steps involving phytoene desaturase (PDS), zeaxanthin desaturase (ZDS), and carotenoid isomerase (CRTISO) [24,25]. Lycopene undergoes cyclisation to form β-carotene, which is then hydroxylated by β-carotene hydroxylase (BCH) to zeaxanthin [24,25], as shown in Figure 3.

Zeaxanthin may be epoxidated by zeaxanthin epoxidase (ZEP), resulting in the formation of violaxanthin. Finally, the conversion of violaxanthin into neoxanthin represents a pivotal branching point in the metabolic process [24,25,26].

The synthesis of Fx from neoxanthin involves a series of reactions, including ketolation at C8, acetylation at C3′, and keto-enol tautomerisation [24,25]. Recent studies have identified carotenoid isomerase 5 (CRTISO5) as the enzyme catalysing the final step, converting phaneroxanthin directly into Fx via a unique hydration mechanism [24,25,27,28] (see Figure 2).

Fx chemical structure has the IUPAC name: 3-hydroxy-4- [(3E,5E,7E,9E,11E,13E,15E)-18-(4-hydroxy-2,2,6-trimethyl-7-oxabicyclo [4.1.0]heptan-1-yl)-3,7,12,16-tetramethyl-17-oxooctadeca1,3,5,7,9,11,13,15-octaen-1-ylidene]-3,5,5-trimethylcyclohexyl acetate [29].

Fx chemical characteristics are unique and complex. The compound is structurally characterised by an epoxide ring, multiple hydroxyl groups, a conjugated carbonyl moiety embedded within an extended polyene chain, and a distinctive allene functionality (Figure 1). This allenic bond is relatively rare in naturally occurring carotenoids and contributes to the distinctive reactivity of Fx [30].

Fx also exhibits several chiral centres and conjugated double bonds, contributing, respectively, to its stereochemical complexity and strong absorption in the visible region, particularly around 450 nm, which explains its orange-brown colouration [31]. The combination of unsaturation and oxygen-containing functional groups makes Fx particularly prone to oxidative degradation and isomerisation [8,32]. As a result, biological materials containing Fx are chemically unstable when exposed to environmental stressors such as heat, light, oxygen, and even acidic or basic pH conditions [8,32]. Fx is lipophilic, and its lipophilicity compromises its aqueous solubility, thereby influencing its extraction processes and limiting its bioavailability [9].

Following this summary of Fx, including its natural sources and biosynthetic pathway, it is important to highlight that, despite promising evidence of Fx’s beneficial effects (from in vitro and in vivo studies), clinical research involving Fx in humans remains scarce.

### 2.3. Biological Activities

To date, only one clinical trial has evaluated the effects of Fx alone (without extracts or in combination with other supplements) (NCT03613740). This clinical assay reported that Fx supplementation led to significant reductions in body weight, hepatic fat accumulation, and caused a reduction in inflammatory markers, alongside improvements in metabolic parameters in patients with non-alcoholic fatty liver disease (NAFLD) or obesity, without notable adverse effects [33,34].

This clinical perspective is supported by the wide range of biological activities that have been observed in vitro and in vivo, which justify the growing scientific interest in Fx. Fx has been reported to exhibit a broad range of biological activities, with effective concentrations varying according to the target system and assay conditions.

In in vitro studies (various cancer cell lines), Fx has demonstrated anticancer effects at concentrations ranging from 5 to 50 µM [6,35,36,37,38,39,40,41,42,43,44,45], and antioxidant activity has been detected at concentrations as low as 10 µM [2,46,47]. The anti-inflammatory effects of Fx have been reported to occur within the range of 5–30 µM, in cell-based models [3,4,48].

Antibacterial activity has also been reported, with minimum inhibitory concentrations ranging from 50 to 200 µg/mL [49,50,51]. In addition, the tissue-protective effects of Fx on the skin [52,53], bones [54], eyes [55,56], and liver [57] have been described, typically at concentrations of 10–50 µM in vitro and 0.1–2 mg/kg in vivo.

In vivo studies have confirmed that Fx exerts both anti-obesity and antidiabetic effects, with significant activity observed at doses between 0.2 and 0.4 mg/kg [58,59]. Notably, anti-obesity effects have been consistently demonstrated in animal models following oral administration of Fx at doses ranging from 0.2 to 2 mg/kg/day [5,60,61,62,63].

In developing this work, particular emphasis was placed on sourcing evidence from original research articles rather than relying exclusively on secondary or review-based literature. This approach was employed to provide a more accurate, up-to-date, and experimentally substantiated overview of Fx’s biological activities, addressing a common limitation of previous reviews, which often report effects lacking direct experimental validation.

## 3. Natural Derivatives of Fucoxanthin

Given its intrinsic chemical instability, it is crucial to understand the metabolic transformation of Fx to evaluate its in vivo bioavailability and bioactivity.

Following ingestion, Fx is subjected to extensive metabolism, predominantly within the gastrointestinal tract and liver, generating a range of metabolites with distinct bioactivities [64,65,66].

**Figure 4:**
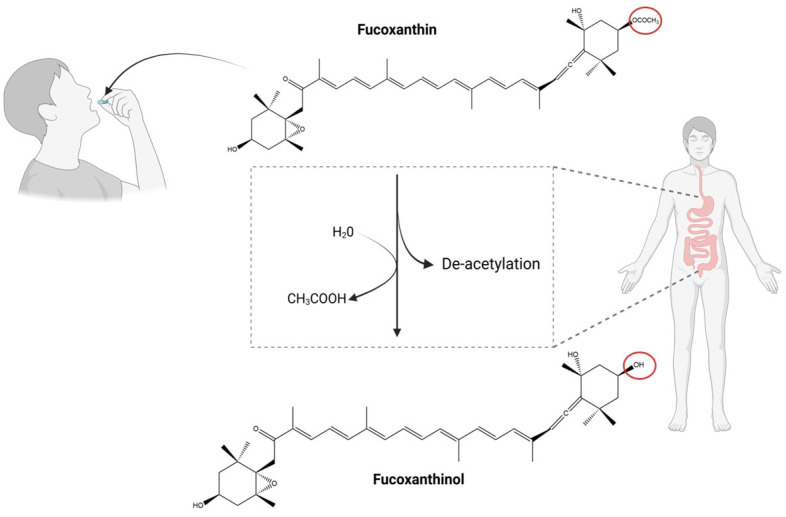
Schematic representation of fucoxanthin (Fx) bioconversion to fucoxanthinol (FxOH) in the gastrointestinal tract. The red forms highlight the structural differences between Fx and its metabolites. Chemical structures were created using ChemDraw Ultra 12.0. Created in BioRender. Nogueira, P. (2025) https://BioRender.com/q6tbtsh (accessed on 13 June 2025).

In the gastrointestinal tract, Fx’s hydrolysis is catalysed by pancreatic enzymes, including lipases and esterases, which function by removing the acetyl group from Fx [64,67] (Figure 4).

This deacetylation process is pivotal in the conversion of Fx into fucoxanthinol [64] (FxOH, Figure 4). The conversion of Fx into FxOH is primarily attributed to secreted digestive enzymes, although the involvement of intestinal microbiota in this process cannot be ruled out.

Once formed, FxOH is absorbed and transported to the liver, where it undergoes further biotransformation via NAD(P)^+^-dependent dehydrogenation/isomerisation reactions [65].

**Figure 5:**
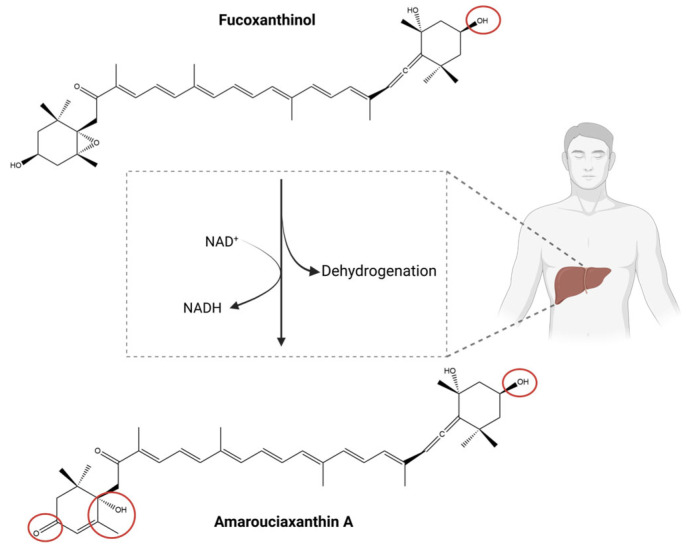
Schematic representation of the metabolic conversion of fucoxanthinol (FxOH) to amarouciaxanthin A following hepatic processing. The red forms highlight the structural differences between fucoxanthin (Fx) and its metabolites. Chemical structures were created using ChemDraw Ultra 12.0. Created in BioRender. Nogueira, P. (2025) https://BioRender.com/npazznz (accessed on 13 June 2025).

The enzymes involved have not yet been definitively identified. This metabolic conversion is catalysed by liver microsomal dehydrogenases, resulting in the formation of amarouciaxanthin A [65] (Figure 5).

Among the resulting metabolites, FxOH and amarouciaxanthin A are recognised as the primary bioactive Fx metabolites, largely responsible for the biological effects associated with Fx [68].

**Figure 6:**
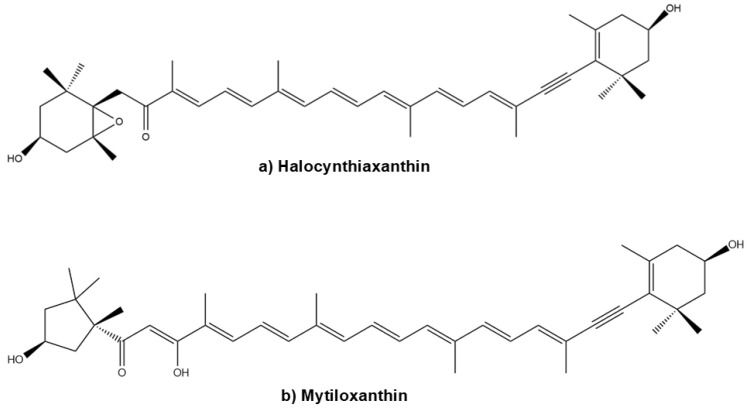
Chemical structures of halocintiaxanthin (**a**) and mitiloxanthin (**b**), two marine xanthophylls identified as metabolites of fucoxanthin (Fx). Despite the structural divergence in the terminal rings, both compounds maintain the characteristic polyene chain. Chemical structures were created using ChemDraw Ultra 12.0.

In addition to the well-characterised compounds described, several other Fx metabolites have been reported in the literature, including halocynthiaxanthin (Figure 6a), and mytiloxanthin [69] (Figure 6b).

These metabolites are the result of species-specific metabolic transformations occurring in various organisms, including mammals, bivalves, tunicates, and hens [69]. Each species exhibits distinct enzymatic pathways, resulting in a diverse set of Fx-metabolites with potentially different biological functions [69]. The interspecies variability observed in Fx metabolism underscores its biochemical complexity and the breadth of its biological effects.

**Figure 7:**
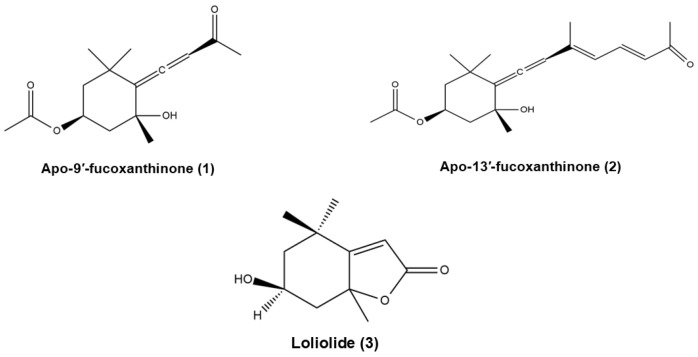
Chemical structures of apo-9′-fucoxanthinone (**1**), apo-13′-fucoxanthinone (**2**) and loliolide (**3**). Chemical structures were created using ChemDraw Ultra 12.0.

Among the known derivatives of Fx, apo-9′-fucoxanthinone (**1**) (Figure 7) is one of the most thoroughly characterised to date. This oxidised metabolite is the result of the oxidative degradation of Fx [70,71]. Naturally, **1** is formed through a combination of spontaneous environmental oxidation but may be formed by chemical processes, including permanganate-mediated reactions [71,72].

In a similar manner, apo-13′-fucoxanthinone (**2**) is produced via oxidative cleavage of Fx, which may occur either under natural conditions or experimentally, through ozonolysis of the purified compound [72].

The isolation and structural characterisation of both **1** and **2** has been achieved through the utilisation of chromatographic and spectroscopic techniques [72].

Another notable degradation product of Fx is loliolide (**3**), which is formed under anoxic or photo-oxidative conditions, particularly in microalgae and macroalgae [73,74,75,76]. The formation of this compound involves specific oxidative cleavage steps and has been documented in a range of marine organisms [73].

The metabolite mytiloxanthin has been reported to present antioxidant activity using a chemical system that mimics physiological conditions, assessing its ability to scavenge singlet oxygen and hydroxyl radicals, as well as its impact on lipid peroxidation. Notably, mytiloxanthin significantly reduced singlet oxygen, hydroxyl radicals, and lipid hydroperoxide (LOOH) formation more effectively than either Fx or FxOH [77].

## 4. Integrated Overview of the Bioactivities of Fx and Its Derivatives

To deepen our understanding of the physiological relevance and therapeutic potential of Fx, its metabolites, and natural derivatives, Table 1 was elaborated to summarise their documented bioactivities.

Understanding the molecular mechanisms underlying the biological activities of Fx’s metabolites is essential to exploring their therapeutic potential. These compounds exert their effects by modulating distinct intracellular signalling pathways, which are closely linked to specific biological activities, as summarised in Table 1.

## 5. Signalling Pathways Modulated by Fx’s Metabolites

The most significant pathways influenced by FxOH and amarouciaxanthin A are the nuclear factor kappa B (NF-κB) [93,94], the nuclear factor erythroid 2-related factor 2 (Nrf2) pathway [95,96]; and the peroxisome proliferator-activated receptor alpha (PPAR-α) pathway [97,98].

The following sub-sections provide a concise overview of each pathway, followed by an analysis of how Fx metabolites modulate their activity and the resulting biological effects.

### 5.1. NF-κB Pathway

NF-κB is a protein complex that functions as a transcription factor, playing a central role in regulating gene expression in response to stimuli such as inflammatory cytokines, microbial products, and cellular stressors [93,94].

The NF-κB family constitutes a group of structurally related proteins, comprising RelA (p65), RelB, c-Rel, p50 (from NF-κB1), and p52 (from NF-κB2), which form homo- or heterodimers. These dimers are stabilised by a conserved Rel homology domain (RHD), which has been demonstrated to mediate both DNA binding and dimerisation [99].

**Figure 8:**
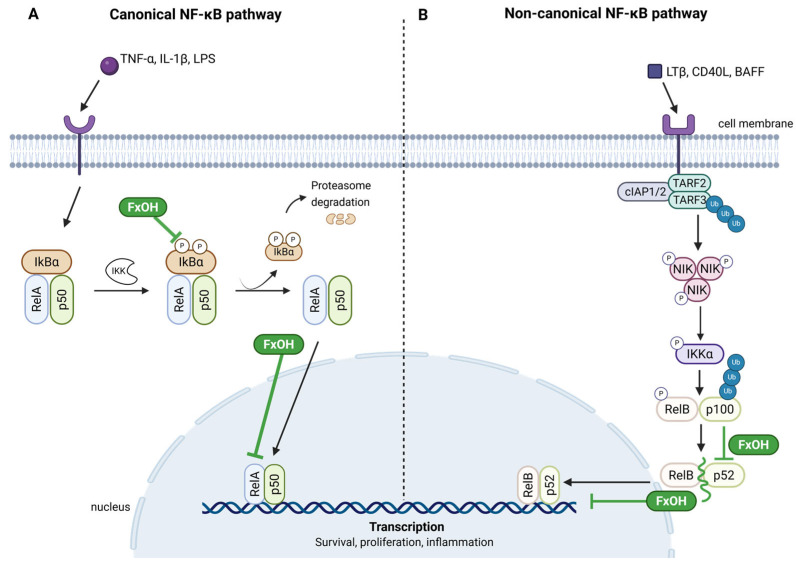
Schematic representation of the canonical (**A**) and non-canonical (**B**) NF-κB signalling pathways and their modulation by fucoxanthinol (FxOH). FxOH has been reported to inhibit the phosphorylation and degradation of IκBα, thereby preventing the nuclear translocation of the RelA/p50 complex in the canonical pathway. Concurrently, FxOH interferes with the processing of p100 into p52, thereby limiting the nuclear activity of the RelB/p52 complex in the non-canonical pathway. The effects contribute to the downregulation of NF-κB-dependent transcription of genes involved in inflammation, survival, and proliferation. (ACO: acyl-CoA oxidase; ACS: acyl-CoA synthetase; Apo-AII: apolipoprotein A-II; Apo-AV: apolipoprotein AV; BAFF: B-cell activating factor; CAP: C-Cbl associated protein; CD40: CD40 ligand; cIAP1/2: cellular inhibitor of apoptosis protein 1/2; CYP4A1: cytochrome P450 family 4 subfamily A member 1; IκBα: inhibitor of NF-κB alpha; IKKα: IκB kinase alpha; IL-1β: interleukin-1 beta; LCAD: long-chain acyl-CoA dehydrogenase; LPL: lipoprotein lipase; LTβ: lymphotoxin beta; ME1: malic enzyme 1; NIK: NF-κB-inducing kinase; NSAIDs: nonsteroidal anti-inflammatory drugs; OLR1: oxidised low-density lipoprotein receptor 1; P: phosphate; PGAR: peroxisome proliferator-activated receptor gamma activator response; PLTP: phospholipid transfer protein; p50/p52/p100: NF-κB subunits; RelA/RelB: v-rel reticuloendotheliosis viral oncogene homologue A/B; RXR: 9-cis-retinoic acid receptor; SCD-1: stearoyl-CoA desaturase-1; TARF2/TARF3: TNF receptor-associated factor 2/3; TNF-α: tumour necrosis factor alpha; UCP-1: uncoupling protein 1; Ub: ubiquitin). Created in BioRender. Nogueira, P. (2025) https://BioRender.com/wl2ogsl (accessed on 13 June 2025).

The NF-κB pathway is activated by extracellular stimuli and is a pivotal regulator of inflammation, immune response, cell survival, and development [93,94,99]. Activation of the NF-κB pathway can proceed via two distinct yet interconnected signalling routes: the canonical and non-canonical pathways [93,94] (Figure 8). Each pathway has been demonstrated to respond to distinct stimuli and to mediate distinct biological outcomes.

The canonical NF-κB signalling pathway is a rapid and extensively regulated response mechanism activated by a wide variety of extracellular stimuli, including pro-inflammatory cytokines (e.g., TNF-α, IL-1β), microbial products (e.g., lipopolysaccharides), and by cellular stress signals [93,100]. Upon stimulation, these signals converge on the IκB kinase (IKK) complex, composed of the catalytic subunits IKKα and IKKβ, along with the regulatory subunit NEMO (also known as IKKγ) [93,94]. Activation of the IKK complex leads to the phosphorylation of IκB proteins, primarily IκBα, which targets them for ubiquitin-mediated proteasomal degradation [93,94]. This degradation event has been shown to liberate NF-κB dimers, which are typically composed of p65 (RelA) and p50 subunits, from their cytoplasmic inhibitors [93,94]. Following their release, these dimers translocate to the nucleus, where they initiate the transcription of a wide array of genes involved in inflammation, immune responses, and cell survival [93,94].

The non-canonical pathway is activated by a more limited set of ligands, predominantly those associated with the tumour necrosis factor (TNF) receptor superfamily, including BAFFR, CD40, and the lymphotoxin β receptor [93] (Figure 8B). The activation of this pathway is dependent on the stabilisation and accumulation of NF-κB-inducing kinase (NIK), which in turn activates IKKα homodimers in a NEMO-independent manner [93,94]. After activation of IKKα, the p100 precursor protein is phosphorylated, thus triggering its partial proteasomal processing into p52. This, in turn, forms a transcriptionally active heterodimer with RelB. The p52/RelB complex subsequently translocates to the nucleus, where it regulates the expression of genes associated with lymphoid organogenesis, immune cell differentiation, and the maintenance of immune homeostasis [93,94].

As described, NF-κB is a central regulator of cell survival and inflammatory responses, and its dysregulation is frequently implicated in the pathogenesis of cancer. Cancer is a multifactorial disease characterised by deregulated cell proliferation and the progressive acquisition of hallmark characteristics that enable tumour initiation, progression, and therapeutic resistance [101,102].

The NF-κB pathway also plays a pivotal role in the processes of inflammation, cell survival, and resistance to apoptosis [93,103]. FxOH has been shown to inhibit the activation of NF-κB by stabilising inhibitor IκBα, thereby preventing NF-κB translocation to the nucleus and reducing the transcription of inflammatory mediators in lipopolysaccharide (LPS)-stimulated microglial and macrophage models [86,89]. Studies have shown that FxOH interferes with a critical step in the canonical NF-κB activation pathway (Figure 8A) by specifically inhibiting the phosphorylation of the p65 subunit at serine 536 (Ser536) [79,80]. This post-translational modification is essential for the nuclear translocation of p65 and the subsequent activation of anti-apoptotic genes such as Bcl-2 [104]. By blocking this phosphorylation, FxOH prevents the nuclear accumulation of p65, thereby impairing its dimerisation with p50 and its binding to DNA. This inhibition significantly reduces the expression of cell survival genes, an effect particularly evident in highly aggressive breast cancer cells, such as the MDA-MB-231 line [79,80,104].

FxOH has also been reported to inhibit the non-canonical NF-κB signalling route (Figure 8B). FxOH interferes with the non-canonical activation by reducing intracellular RelB levels and inhibiting the conversion of p100 into p52, thereby disrupting the formation of the active p52/RelB complex [79,80]. This inhibition results in a reduction in both cytoplasmic and nuclear levels of RelB and p52, which ultimately leads to impaired transcription of genes, namely some involved in cancer cell survival and resistance to apoptosis [79,80,104].

These findings are further corroborated by complementary studies conducted in MCF-7 breast cancer cells, where both FxOH and halocynthiaxanthin exhibited antiproliferative and pro-apoptotic effects [81]. In this study, FxOH was found to be the most effective apoptosis inducer, thereby validating its status as a promising lead compound for the development of novel therapeutic interventions targeting NF-κB-driven malignancies [81].

By downregulating key NF-κB subunits—p65 (canonical), p52, and RelB (non-canonical), simultaneously, FxOH exerts a dual blockade on NF-κB-mediated transcriptional programmes. This coordinated suppression has been demonstrated to disrupt the expression of genes involved in cell survival, proliferation, and inflammation, leading to enhanced apoptosis and reduced viability of breast cancer cells [79,80,81,83].

It is notable that NF-κB signalling supports several established hallmarks of cancer, including resistance to cell death, sustained proliferative signalling, and tumour-promoting inflammation [94,105]. Therefore, FxOH’s ability to target both arms of this pathway underscores its potential as a multifaceted anticancer agent.

### 5.2. Nrf-2 Pathway

Nrf2 is a protein that functions as a transcription factor, thereby regulating the expression of numerous genes involved in the cellular antioxidant and detoxification response [106,107,108]. In addition to playing a pivotal role in the protection against oxidative stress, Nrf2 also participates in metabolic processes, modulates the inflammatory response, and contributes to the maintenance of cellular and tissue homeostasis [107,108].

Nrf2 is a basic leucine zipper transcription factor that regulates the expression of a wide range of antioxidant, detoxifying, and cytoprotective enzymes, thereby enhancing cell survival under environmental or metabolic stress conditions [109].

**Figure 9:**
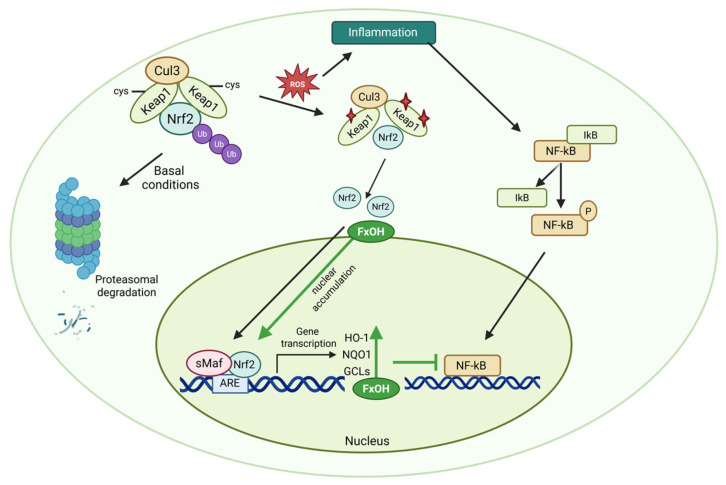
Proposed dual modulatory mechanism of fucoxanthinol (FxOH) on the Nrf2 and NF-κB pathways. Under oxidative or inflammatory stress, FxOH has been observed to promote the dissociation of Nrf2 from Keap1, thereby enhancing its nuclear accumulation and transcriptional activation of antioxidant genes, including HO-1, NQO1, and GCLs. Concurrently, FxOH inhibits NF-κB signalling by stabilising IκBα, thereby preventing NF-κB nuclear translocation and suppressing the expression of pro-inflammatory mediators. Red star symbols denote ROS-induced modifications of Keap1 cysteine residues, leading to Nrf2 dissociation. (ARE: antioxidant response element; Cul3: cullin 3; GCLs: glutamate-cysteine ligase; HO-1: heme oxygenase 1; IκB: inhibitor of NF-κB; Keap1: kelch-like ECH-associated protein 1; NQO1: NAD(P)H:quinone oxidoreductase 1; Nrf2: nuclear factor erythroid 2–related factor 2; P: phosphate; ROS: reactive oxygen species; sMaf: small Maf protein; Ub: ubiquitin). Created in BioRender. Nogueira, P. (2025) https://BioRender.com/qpcjdl1 (accessed on 13 June 2025).

In conditions of homeostatic balance, Nrf2 is maintained within the cytoplasm by Kelch-like ECH-associated protein 1 (Keap1), which signals its targeting for ubiquitin-mediated proteasomal degradation [109] (Figure 9). Upon oxidative or electrophilic challenge, modifications to critical cysteine residues in Keap1 disrupt this interaction, allowing Nrf2 to accumulate and translocate into the nucleus [109].

The importance of the Nrf-2 pathway is widely recognised, extending to various tissues in the body. The influence of this factor is significant in several areas, including metabolism and protection against neurodegenerative and chronic diseases [107,108,109]. The Nrf2 pathway has been shown to play a central role in cellular defence against oxidative and electrophilic stress [107,109]. In the nucleus, Nrf2 dimerises with small Maf proteins and binds to antioxidant response elements (AREs) in the promoter regions of target genes. This activation process has been shown to promote the transcription of cytoprotective enzymes, including heme oxygenase-1 (HO-1), NAD(P)H:quinone oxidoreductase-1 (NQO1), glutathione S-transferase (GST), and superoxide dismutase (SOD) [110].

Beyond the regulation of redox processes, Nrf2 also modulates inflammation. Nrf2 exerts anti-inflammatory effects via two complementary mechanisms: (1) indirectly, by reducing reactive oxygen species (ROS) and inducing HO-1, which interferes with NF-κB nuclear translocation, and (2) directly, by suppressing RNA polymerase II recruitment to pro-inflammatory gene promoters [110,111]. This bidirectional interplay forms a feedback loop that helps resolve inflammation and maintain immune equilibrium [112].

FxOH functions as a dual modulator of the Nrf2 pathway (Figure 9). Studies have demonstrated that FxOH enhances the nuclear accumulation of Nrf2, thereby upregulating enzymes such as HO-1 and NQO1 [89,113,114]. The upregulation of these enzymes has been shown to boost the cellular antioxidant capacity and contribute to the suppression of pro-inflammatory signalling.

Together, these data highlight FxOH as a multifunctional compound capable of preventing pro-inflammatory cascades and potentiating cytoprotective responses through the orchestrated regulation of Nrf2, NF-κB, and PPAR-α pathways, as elucidated in Table 1.

### 5.3. PPARs Signalling Pathway

Peroxisome proliferator-activated receptors (PPARs) represent a subfamily of nuclear receptors that act as transcription factors, regulating gene expression involved in key physiological processes such as lipid, carbohydrate, and protein metabolism, as well as inflammation, cellular differentiation, and tissue development [115,116].

There are three main isoforms—PPARα, PPARβ/δ, and PPARγ—each with distinct tissue distributions and biological functions [115,116].

PPARs are activated by binding to endogenous ligands, such as free fatty acids and eicosanoids, or synthetic agonists like fibrates (PPARα) and thiazolidinediones (PPARγ) [117,118,119]. Notably, PPARγ plays a central role in lipid homeostasis, glucose regulation, and adipocyte differentiation, and is highly expressed in adipose tissue [115,116].

**Figure 10:**
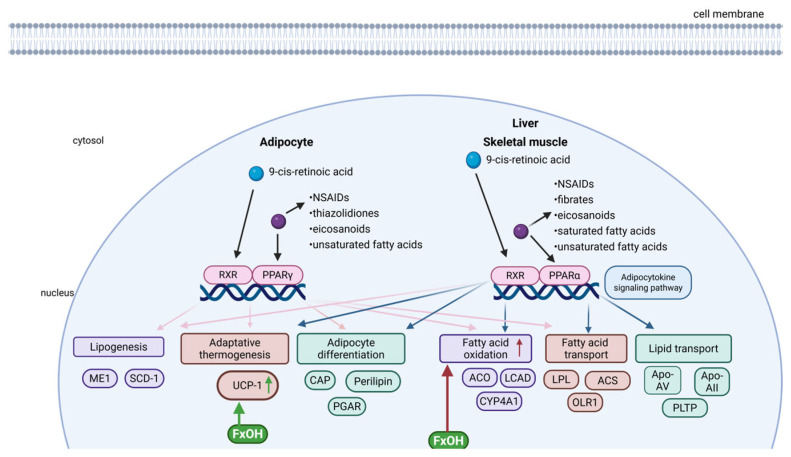
Fucoxanthinol (FxOH) regulation of lipid metabolism and thermogenesis via PPAR pathways in adipose tissue. FxOH activates PPARγ and PPARα signalling, leading to upregulation of UCP-1 in adipocytes and enzymes involved in fatty acid oxidation in hepatic and muscle tissue. These actions highlight the dual metabolic and anti-obesity potential of FxOH. The green arrows adjacent to FxOH and UCP-1 represent effects experimentally demonstrated, whereas the red arrow indicates indirect mechanisms that are not yet fully elucidated. (ACO: acyl-CoA oxidase; ACS: acyl-CoA synthetase; Apo-AII: apolipoprotein A-II; Apo-AV: apolipoprotein AV; CAP: C-Cbl associated protein; CYP4A1: cytochrome P450 family 4 subfamily A member 1; LCAD: long-chain Acyl-CoA dehydrogenase; LPL: lipoprotein lipase; ME1: malic enzyme 1; NSAIDs: nonsteroidal Anti-Inflammatory drugs; OLR1: oxidised low-density lipoprotein receptor 1; PGAR: peroxisome proliferator-activated receptor gamma activator response; PLTP: phospholipid transfer protein; RXR: 9-cis-retinoic acid receptor; SCD-1: stearoyl-CoA desaturase-1; UCP-1: uncoupling protein 1). Created in BioRender. Nogueira, P. (2025) https://BioRender.com/ranvwny (accessed on 13 June 2025).

Upon PPAR activation, they heterodimerize with the 9-cis-retinoic acid receptor (RXR) and translocate to the nucleus, where they bind to specific DNA sequences known as peroxisome proliferator response elements (PPREs) (Figure 10). This process modulates the transcription of genes involved in metabolic and inflammatory pathways [118,119].

Even though Fx and FxOH are not classified as specific inhibitors of PPARs, evidence from cellular and animal studies indicates that both compounds can modulate PPAR-related metabolic activity [5,85,86]. PPAR modulation by Fx appears to occur predominantly through indirect mechanisms, including the regulation of PPAR-responsive gene expression and interaction with associated signalling pathways. In macrophages, FxOH has been demonstrated to exert immunomodulatory effects by targeting the NAAA (N-acylethanolamine acid amidase)–PEA (palmitoylethanolamide)–PPARα axis. Specifically, FxOH reverses the LPS-induced downregulation of PEA and suppresses the expression of pro-inflammatory cytokines [86]. This effect is abolished in the presence of PPAR-α inhibition, highlighting the dependence of FxOH on this receptor.

Moreover, FxOH has been documented to exert a regulatory influence on adipocyte differentiation, accomplished through the modulation of PPARγ signalling [85]. In vitro studies have demonstrated that FxOH exerts its function by downregulating adipogenic gene expression, thereby reducing lipid accumulation during the process of preadipocyte differentiation [85].

PPARs are particularly relevant in the context of metabolic disorders. Treatments targeting PPARs enhance insulin sensitivity, improve glucose and lipid profiles, promote healthy adipocyte differentiation, and mitigate chronic low-grade inflammation, all of which contribute to restoring metabolic homeostasis [116,120,121]. These multifactorial effects underscore the therapeutic potential of PPARγ agonists not only in type 2 diabetes and dyslipidemia, but also in the treatment of obesity and associated metabolic dysfunctions [116,120].

Obesity is a complex, multifactorial condition characterised by excessive accumulation of adipose tissue resulting from an imbalance between energy intake and expenditure [122]. The condition has been linked to an elevated probability of metabolic disorders, encompassing type 2 diabetes, cardiovascular disease, and specific forms of cancer [123,124,125]. It has emerged as a significant global health concern, driven by its increasing prevalence [126].

Literature suggests that obesity may not be exclusively attributable to excessive caloric intake; rather, it is also driven by chronic low-grade inflammation within adipose tissue [127,128,129]. As adipose tissue expands, it undergoes cellular stress and recruits immune cells, particularly macrophages [130]. These cells secrete pro-inflammatory cytokines, such as TNF-α, IL-6, and MCP-1 [130,131,132]. It has been demonstrated that this inflammatory environment disrupts insulin signalling and metabolic homeostasis, thereby perpetuating further weight gain and metabolic dysfunction [131,133]. Consequently, inflammation is both a consequence and a driver of obesity, creating a vicious cycle that underlies many obesity-related complications.

Given the well-established link between chronic inflammation and obesity, FxOH has emerged as a promising anti-obesity agent by simultaneously targeting metabolic and inflammatory pathways [59]. On the metabolic level, FxOH has been observed to modulate the expression of genes involved in lipid metabolism, with notable upregulation of uncoupling protein-1 (UCP-1) in white adipose tissue [59,85]. This upregulation enhances thermogenesis and fatty acid oxidation, collectively contributing to decreased lipid accumulation and improved energy expenditure [85] (Figure 10).

In parallel, FxOH exhibits potent anti-inflammatory properties within adipose tissue. It suppresses the production of key pro-inflammatory cytokines, such as TNF-α and IL-6, which are known mediators of insulin resistance and metabolic dysfunction in obesity [59]. By attenuating local inflammation, FxOH helps to restore insulin sensitivity and supports overall metabolic health [59].

Together, these dual actions—improving metabolic function and reducing inflammation—highlight the therapeutic potential of FxOH for obesity management, especially in cases where inflammation is a central driver of metabolic disturbances [59].

## 6. Challenges to Obtain Synthetic Fx and Fx Derivatives

Historically, the complete synthesis of Fx has presented significant challenges, primarily due to its conjugated polyene chain, the presence of an allene molecule and a conjugated carbonyl group. These features all contribute to Fx’s structural complexity, as discussed above.

**Figure 11:**
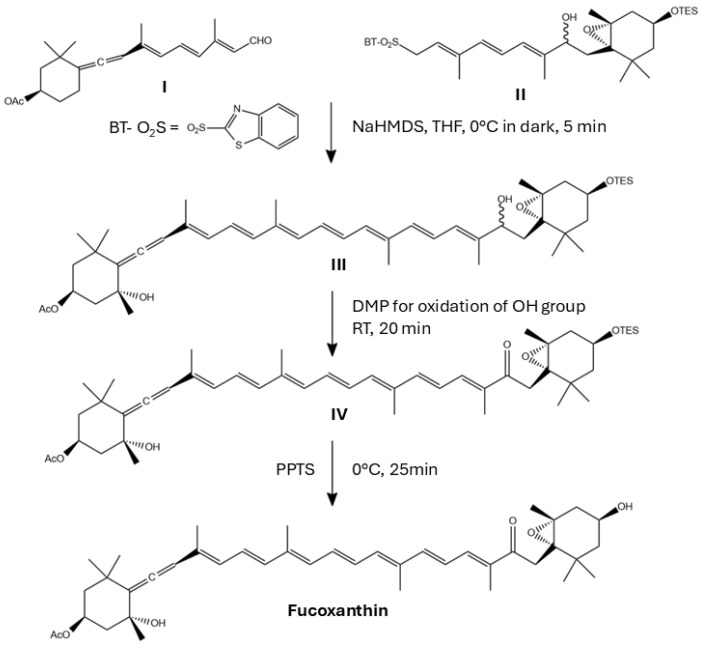
Proposed synthetic pathway of fucoxanthin (Fx) as reported by Gupta et al. Key reagents: NaHMDS: sodium bis(trimethylsilyl)amide; DMP: Dess–Martin periodinane; PPTS: pyridinium p-toluenesulfonate. Adapted from [138]. Chemical structures were created using ChemDraw Ultra 12.0.

Early synthetic approaches were often time-consuming and laborious, requiring precise control of stereochemistry and functional group positioning [30,134,135]. The ability to exercise control over regio- and stereoselectivity is of particular importance, given the high sensitivity of the biological properties of Fx derivatives to the position and orientation of these chemical changes [85,135]. Advances in synthetic methodologies, including microwave-assisted synthesis, photoreduction, and the use of specialised catalysts, have contributed to improvements in yields and selectivity [136,137]. Moreover, the integration of chemical synthesis with biocatalytic approaches offers a promising solution for the efficient and sustainable production of structurally diverse and biologically potent Fx derivatives [136]. Recent advances in stereoselective methodologies and the strategic utilisation of key intermediates have significantly improved the synthesis efficiency and yield [85,135]. The best-known synthetic method to date was proposed by Gupta et al. [138] (Figure 11).

Building on recent advances in synthetic chemistry, a small number of Fx derivatives have been developed with the aim to enhance their chemical stability, bioavailability, and functional versatility—persistent challenges that continue to hinder their full therapeutic potential. The molecule’s interaction with biological systems is optimised by structural modifications, including esterification with bile acids or the incorporation of specific functional groups.

Table 2 summarises the Fx derivatives described so far, highlighting their structural characteristics, origin, experimental or computational models where they were tested, and the predicted or observed biological effects.

Isofucoxanthinol (**4**) and fucoxanthinol-hemiketal (**5**) are the chemical derivatives of Fx, obtained through the process of alkaline treatment using 5% potassium hydroxide (KOH) in methanol [139]. It is evident that **4** retains the distinctive allenic bond and acetyl group of the parent compound [139]. However, it adopts an open-chain configuration, characterised by a hydroxylated polyene backbone, multiple conjugated double bonds, and a terminal keto group. Conversely, **5** undergoes intramolecular cyclisation, forming a hemiketal ring through the interaction between its keto and hydroxyl groups, while also preserving the allenic moiety [139]. The structural differences between these two types of compounds, namely the linear configuration observed in 4 and the cyclic configuration present in **5**, have been shown to significantly influence their physicochemical properties. This influence can be seen in properties such as polarity and stability, which are reflected in the divergent retention times of the compounds as measured by HPLC analyses [139].

The derivatives lithocholylfucoxanthin (**6**), lithocholylfucoxanthinol (**7**), lithocholylfucoxanthin levulinate (**8**), and levulinate lithocholylfucoxanthinol (**9**) represent a series of novel Fx-based conjugates designed to modulate hydrophobicity and interactions with bile acids [140]. The hydrophobic nature of lithocholic acid has been demonstrated to enhance the absorption and stability of Fx within the gastrointestinal tract, thereby increasing its bioavailability [140]. This is a significant improvement over the bioavailability of natural Fx, which has been shown to be limited. Concurrently, these conjugates capitalise on both Fx’s capacity to promote fat burning and bile acids’ function in metabolic signalling, thus offering a dual-action approach that targets weight management and associated metabolic disorders [141,142]. The syntheses of these compounds involve the esterification of Fx or FxOH with lithocholic acid, a highly hydrophobic secondary bile acid [140].

The levulinyl group has been demonstrated to enhance hydrophobicity and to function as a cleavable protecting group, thereby enabling selective deprotection and structural diversification [140]. These modifications substantially alter the physicochemical behaviour of the conjugates in bile salt/phospholipid mixed micelle systems [140]. This demonstrates how strategic chemical derivatisation of Fx can influence its amphiphilic profile, potentially enhancing its solubility, stability, and bioavailability, which are key parameters for its functional and therapeutic applications [140].

## 7. Bioactivity of Synthetic Fx Derivatives

As presented in Table 2, two semi-synthetic Fx derivatives have shown promising biological activity. Compounds **4** and **5** have shown notable anti-obesity effects in high-fat diet-fed mice [139]. Both compounds decreased body weight gain and fat accumulation. Both compounds were administered at concentrations of 0.1% (*w*/*w*) in the animal’s diet, resulting in reduced body weight gain and reduced fat accumulation [139]. At the molecular level, particularly **5** was shown to modulate lipid metabolism by downregulating lipogenic genes and upregulating fatty acid oxidation genes, in both adipose tissue and skeletal muscle. These findings suggest their potential to combat obesity by limiting lipid uptake and enhancing fat metabolism [139].

A significant challenge for the synthesis of more active and selective Fx derivatives is the complexity and variability of metabolic pathways across different organisms. As previously mentioned, each species possesses distinct enzymatic routes, leading to unique profiles of metabolites with diverse biological functions. This interspecies variability, while technically challenging, can also be viewed as a strategic opportunity within the field of biotechnology.

By mapping and comparing the metabolic pathways involved in Fx transformation across a range of organisms, including mammals, bivalves, tunicates and birds, it becomes possible to identify points of convergence and key enzymes responsible for the generation of the most biologically relevant metabolites. This knowledge paves the way for the application of metabolic engineering strategies, including the modification of model microorganisms (e.g., yeast or bacteria) or the use of non-conventional organisms that share metabolic pathways like those of humans. Such an approach has the potential to facilitate the targeted and sustainable production of bioactive metabolites in controlled systems such as bioreactors. However, turning this vision into reality will require comprehensive, multidisciplinary research involving comparative metabolomics, enzyme characterisation and metabolic flux analysis. This is because the underlying enzymatic networks are still only partially understood, making the engineering of Fx pathways a substantial scientific and technical challenge.

## 8. Structure-Activity Relationship (SAR) Studies

A comprehensive understanding of the influence that structural features exert on the biological activities of Fx and its derivatives is important for the purpose of enabling the rational design of more potent and stable compounds. SAR studies facilitate the identification of key molecular modifications that enhance or attenuate specific bioactivities, thereby offering valuable insight into the functional roles of individual chemical groups.

In Fx and its derivatives, structural variations—such as modifications of the allenic bond, epoxide moiety, hydroxyl substituents, and the length or degree of saturation of the polyene chain—have been linked to distinct antioxidant, anti-inflammatory, anti-obesity, and anticancer activities. Elucidation of these associations is imperative for the development of more potent, selective, and metabolically stable analogues.

The antioxidant activity of Fx is largely attributed to its unique chemical structural features—most notably, the presence of an allenic bond at carbon C-7′ [143,144]. This rare motif among carotenoids, in combination with oxygenated functional groups such as epoxides and hydroxyls, enhances the delocalisation of electrons and increases the compound’s reactivity towards ROS [145]. These structural elements synergistically contribute to an efficient radical-scavenging capacity, thereby mitigating oxidative stress [145].

Fx has been shown to exert anti-inflammatory and antidiabetic effects by targeting enzymes and signalling pathways implicated in metabolic dysregulation [146,147]. These activities are closely linked to its structural features, particularly the extended polyene chain and the presence of hydroxyl and carboxyl groups, which facilitate binding to molecular targets such as aldose reductase and inflammatory mediators, thereby contributing to improved glucose regulation and reduced inflammatory signalling [148]. In particular, the allenic bond and epoxide moiety have been implicated in pivotal roles concerning the suppression of pro-inflammatory cytokines such as TNF-α and IL-6 in adipose and immune cells [149]. The metabolic conversion of Fx into FxOH and amarouciaxanthin A further influences these outcomes, with structural modifications—especially in hydroxylation—modulating their potency and specificity [65].

Antitumor activity is a pivotal bioactivity of Fx and its primary metabolites, which involves the induction of apoptosis, the disruption of mitochondrial function, and the modulation of cancer cell metabolism [150]. These effects are associated with the presence of oxygenated functional groups in the molecular structure of Fx, which enable interactions with intracellular redox systems and influence signalling pathways related to cell survival and oxidative balance [151,152]. Moreover, mechanistic studies in breast cancer models have demonstrated that the metabolite FxOH exerts antitumor effects, in part, through the NF-κB signalling pathway [79,80,89]. The activation of Nrf2 by FxOH also contributes to its antitumor effects by increasing the expression of enzymes that protect the cell against oxidative stress and help eliminate carcinogenic substances [80,111,153]. Supporting these observations, in vitro studies using RAW264.7 macrophages showed that both Fx and FxOH can directly scavenge peroxyl radicals (ROO•) and activate the Nrf2–ARE pathway, leading to increased nuclear Nrf2 and HO-1 expression [154]. Moreover, FxOH produced stronger Nrf2/HO-1 responses than Fx at low concentrations (2.5–10 µM), suggesting that this metabolite presents greater potency in redox regulation [154]. These findings provide mechanistic evidence linking radical scavenging to Nrf2 activation and support the concentration-dependent dual activity of both Fx and FxOH, being cytoprotective at low and pro-apoptotic at higher concentrations [154]. In HBV-infected cells, activation of the Nrf2 pathway has been implicated in the cytoprotective and antiviral actions of Fx. Stimulation of this pathway led to a marked reduction in HBV pregenomic RNA levels, accompanied by an upregulation of Nrf2 and its downstream effector HO-1 [155]. In addition, FxOH inhibits the NF-κB inflammatory pathway (see Figure 9), preventing its entry into the nucleus and the activation of pro-inflammatory genes. The dual action of activating Nrf2 and blocking NF-κB contributes to the inhibition of the inflammatory environment that promotes tumour development.

**Figure 12:**
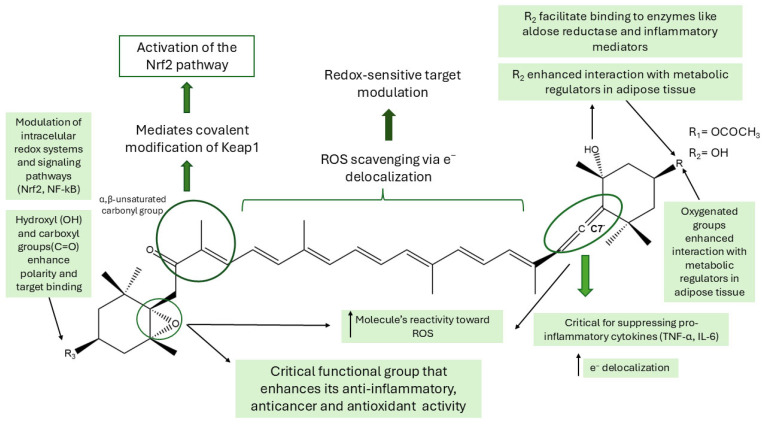
Structure-activity relationships (SAR) scheme of fucoxanthin (Fx), highlighting the main functional groups and their contributions to intracellular redox modulation, Nrf2 pathway activation, suppression of inflammatory cytokines (such as TNF-α and IL-6) and metabolic interactions in adipose tissue. The structural features conserved throughout the molecule’s backbone are associated with anti-inflammatory, antioxidant, anticancer and anti-obesity properties. The chemical structure was created using ChemDraw Ultra 12.0.

Fx and its main metabolites—FxOH, halocynthiaxanthin, and amarouciaxanthin A—share a critical structural motif: the α,β-unsaturated carbonyl group (Figure 12). This moiety functions as a Michael acceptor, allowing these molecules to form covalent bonds with nucleophilic residues, particularly the thiol groups of cysteine residues in regulatory proteins [47]. A notable target is the Keap1 protein, a cytosolic regulator of the Nrf2 signalling pathway. By covalently modifying specific cysteine residues in Keap1, these compounds promote the release and nuclear translocation of Nrf2, thereby activating the expression of antioxidant and cytoprotective genes.

The anti-obesity activity of Fx is primarily attributed to the allenic bond and two hydroxyl groups at the carotenoid end of its polyene chain [144]. These oxygenated groups have been implicated in the enhanced interaction with metabolic regulators in adipose tissue, thereby promoting UCP1 expression in white adipose tissue [142]. This, in turn, has been shown to stimulate mitochondrial fatty acid oxidation and thermogenesis [142], increasing energy expenditure, reducing lipid accumulation, and modulating adipocyte differentiation and lipid metabolism, collectively reinforcing Fx’s anti-obesity [77,142].

Finally, the biotransformation of Fx into FxOH and, subsequently, into amarouciaxanthin A retains several key structural features—particularly hydroxyl groups—that are essential for maintaining or even enhancing specific bioactivities. This metabolic continuity underscores the significance of conserved structural motifs, not only in the parent compound but also in its active metabolites. The structural modifications discussed are illustrated in Figure 12.

**Figure 13:**
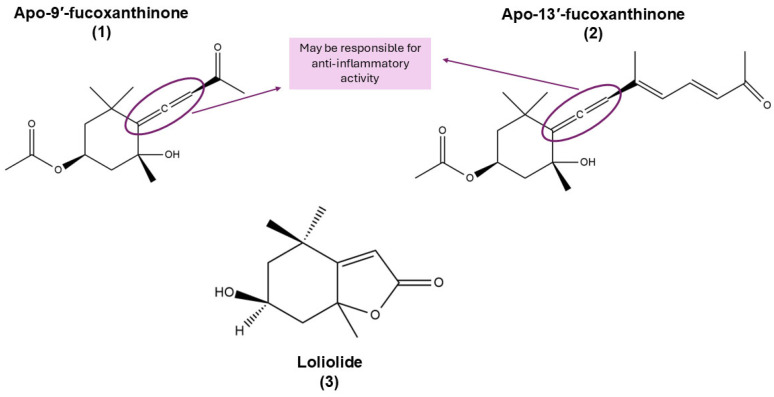
Chemical structures of apo-9′-fucoxanthinone (**1**), apo-13′-fucoxanthinone (**2**), and loliolide (**3**), natural derivatives of fucoxanthin (Fx). The allenic bond, highlighted in compounds **1** and **2**, may be implicated in their anti-inflammatory activity. Chemical structures were created using ChemDraw Ultra 12.0.

Among the natural derivatives (shown in Section 3), compounds **1** and **2** are distinguished by their pharmacological potential, which is a consequence of their unique norisoprenoid structures and the presence of an allenic bond (Figure 13). This bond, shared by both compounds, has also been identified in Fx metabolites such as FxOH and amarouciaxanthin A, and has been proposed as a potential pharmacophore responsible for their anti-inflammatory activity. It is posited that the presence of the allenic bond may be pivotal in the inhibition of NF-κB and Nrf2 signalling pathways, which play pivotal roles in the inflammatory response, based on the available data from FxOH and amarouciaxanthin A.

In contrast, loliolide—a lactomonoterpenoid resulting from the oxidative degradation of Fx—does not possess this bond, which may explain its distinct mechanism of action, primarily associated with antioxidant and indirect anti-inflammatory effects. In view of the structural divergence from other compounds, it is not yet possible to establish a clear structure-activity relationship for loliolide.

Among the synthetic derivatives, compounds **8** and **9** are, to date, the only ones for which experimental evaluation of biological activity has been conducted, revealing remarkable anti-obesity effects. It is noteworthy that both compounds retain the allenic group found in Fx and FxOH, a structural motif associated with anti-inflammatory activity, as illustrated in Figure 12. These findings provide further support for the hypothesis that specific structural features, such as the allenic bond, play a critical role in modulating inflammatory pathways. This underscores their significance in the rational design of Fx-based synthetic derivatives with augmented therapeutic potential. In view of the structural evidence presented, further in vitro studies are imperative to directly assess the anti-inflammatory properties of these compounds.

Fx is structurally distinct from other carotenoids, such as β-carotene and astaxanthin, due to the presence of a unique allenic bond and an epoxide group. These uncommon moieties are critical determinants of its diverse and potent bioactivities, contributing to its functional versatility across various biological systems [146].

A comprehensive understanding of the SAR of Fx and its metabolites is therefore of great value, as it provides valuable insights into the molecular features that underpin their biological functions. Such knowledge not only elucidates their mechanisms of action but also supports the rational development of more stable and efficacious analogues. It is anticipated that forthcoming studies on the use of SAR will prove instrumental in the full realisation of the therapeutic potential of these compounds, which are derived from the sea. This is particularly true of studies combining synthetic chemistry with computational modelling.

## 9. Conclusions

This review examined the relevance of the potential bioactivities of Fx and its metabolites, exploring their trajectory from biosynthesis in marine organisms to their biological activity in human cells. The evidence presented here demonstrates that these compounds modulate critical pathways involved in inflammation, oxidative stress, and lipid metabolism—namely, NF-κB, Nrf2, and PPAR—supporting their multifunctional profile as anti-inflammatory, antioxidant, and antitumour agents.

Nevertheless, critical obstacles persist and must be addressed. The progression of Fx and its derivatives toward clinical applications is constrained by their low physicochemical stability and bioavailability, which reinforces the necessity for innovative pharmaceutical formulations and targeted delivery approaches.

The paucity of mechanistic studies, particularly in physiologically relevant in vitro and in vivo models, still poses a significant challenge to the robust pharmacological validation required for clinical translation. Advancing semi-synthetic approaches and elucidating molecular targets within signalling networks are imperative subsequent steps in optimising these compounds for therapeutic use.

By integrating up-to-date insights into the structural properties, metabolism, and bioactivities of fucoxanthin and its derivatives, this work contributes to a more robust scientific framework for the rational design of new therapeutic interventions. It draws attention to the need for further research to fully elucidate the mechanisms of action of these compounds and improve their pharmacokinetic properties. Despite existing limitations, the evidence gathered to date robustly supports the therapeutic relevance of marine-derived compounds, which can effectively modulate key intracellular pathways—namely those involved in inflammation, oxidative stress, and metabolic regulation—offering promising avenues for the treatment of several diseases with unmet medical needs.

## Figures and Tables

**Table 1:** Cell-based evidence of the biological effects of fucoxanthin metabolites and natural derivatives.

Compound	Compound Type	Bioactivity	Model (Cell Line)	Effect
Fucoxanthinol (FxOH)	Metabolite	Anticancer	Saos-2	Induces apoptosis through activation of caspases-3,-8 and 9 [78] Inhibits cell viability [78] Inhibits cell migration and invasion [78] Inhibits AP-1 activation [78]
			MCF-7 and MDA-MB-231	Inhibits viability in MCF-7 and MDA-MB-231 cells [79,80] Induces apoptosis in MCF-7 and MDA-MB-231 (increased Annexin V signal) [79,80] Reduces SOX9 expression in MDA-MB-231 cells [80] Decreases levels of necrosis [80] Inhibits nuclear accumulation of NF-κB components (p65, p52, RelB) in MDA-MB-231 cells [79,80] Induces DNA fragmentation in MCF-7 cells [81]
			DLD-1	Induces anoikis [82] Inhibits EMT [82]
			HCT116 and HT-29	Induces apoptosis via NF-κB inhibition and IAP suppression [83]
			Caco-2 SW620 DLD-1 WiDr	Reduces cell viability [43,81] Induces DNA fragmentation [81]
			HL-60	Reduces cell viability [81] Induces apoptosis, chromatin condensation and nuclear degradation (DNA damage) [81] Reduces Bcl-2 protein levels [81]
			Raji Daubi BJAB Ramos BJAB L428 KM-H2 HDLM-2 L540	Reduces cell viability [84] Causes G1 cell cycle arrest [84] Induces apoptosis [84]
		Anti-obesity	3T3-L1	Down-regulates PPARϒ [59,85]
		Anti-Inflammatory	RAW264.7 3T3-F442A Hepa-1-6 cells	Reduces production of TNF-α, IL-6, IL-1β and NO [59,86,87,88]
			BV-2	Inhibits LPS-induced inflammatory mediators (iNOs, NO, PGE-2 and COX-2) [89]
		Antioxidant	SH-SY5Y	Reduces ROS formation [2] Increases intracellular GSH through the Nrf2/Keap1/ARE pathway [2]
		Neuroprotective	SH-SY5Y	Preserves neuronal protection against toxicity caused by AβO and 6-OHDA [2]
Halocynthiaxanthin	Metabolite	Anticancer	HL-60	Reduces cell viability [81] Induces apoptosis, chromatin condensation and nuclear degradation (DNA damage) [81] Reduces Bcl-2 protein levels [81]
			MCF-7	Reduces cell viability [81] Induces DNA fragmentation [81]
			Caco-2 cells	Reduces cell viability [81] Induces DNA fragmentation [81]
Amarouciaxanthin A	Metabolite	Anti-inflammatory	Hepa-1-6 cells	Suppresses chemokine production in TNFα-stimulated liver cells [88]
Apo-9′-fucoxanthinone (**1**)	Derivative	Anti-inflammatory	BMDMs and BMDCs RAW 264.7	Inhibits production of pro-inflammatory cytokines (IL-12 p40, IL-6 e TNF-α) and ERK1/2 phosphorylation in macrophages and dendritic cells [90] NO/iNOS/COX-2 regulation, inhibition of NF-κB and JNK/ERK pathways in zebrafish and RAW 264.7 cells [91]
		Anticancer	Caco-2 cells HBEC2 cells	Inhibits cell proliferation [72] Reduces oxidative stress, DNA damage, and chronic inflammation [71]
Apo-13-fucoxanthinone (**2**)	Derivative	Anticancer	Caco-2 cells	Exhibits antiproliferative effects on cancer cells (lower compared to other fucoxanthin derivatives such as apo-9′-fucoxanthinone) [71]
3-hydroxy-DHA (loliolide) (**3**)	Derivative	Anti-inflammatory	RAW 264.7	Suppresses NO production in LPS-stimulated RAW264.7 cells [92] Supresses IL-1β, IL-6, TNF-α, PGE2 COX-2, and iNOS production in LPS-induced cells [75]

Legend: 3T3-F442A: murine preadipocytes; 3T3-L1: mice adipocyte cells; AP-1: activator Protein-1; ARE: antioxidant response elements; AβO: Aβ oligomers; Bcl-2: B-cell lymphoma 2; BMDCs: dendritic cells; BMDMs: bone marrow-derived macrophage; BV-2: microglial cell; Caco-2: human colorectal adenocarcinoma cells; COX-2: cyclooxygenase-2; DLD-1: colorectal adenocarcinoma cells; DLD-1: human colorectal cancer cell line; EMT: epithelial–mesenchymal transition; ERK: extra-cellular signal-regulated kinases; GSH: Glutathione; HBEC2 cells: human bronchial epithelial cells; HCT116 and HT-29: human colorectal cancer cell line; Hepa-1-6 cells: murine hepatoma cell line; HL-60: human leukaemia cells; IAP: inhibitor of apoptosis proteins; IL-12 p40: interleukin12 p40; IL-1β: interleukin-1β; IL-6: interleukin-6; iNOs: inducible nitric oxide synthase; JNK: c-Jun N-terminal cinase; Keap1: kelch-like ECH-associated protein 1; LPS: lipopolysaccharide; MCF-7 and MDA-MB-231: human breast cancer cells; NF-κB: nuclear factor kappa B; NO: nitric oxide; Nrf2: nuclear factor erythroid 2-related factor 2; PGE2: prostaglandin E2; PPARϒ: peroxisome proliferator-activated receptor gamma; Raji, Daubi, BJAB, Ramos, BJAB L428, KM-H2, HDLM-2 and L540: Lymphoma cell lines; RAW264.7: macrophage cell line; ROS: reactive oxygen species; Saos-2: human osteosarcoma cell line; SH-SY5Y: human neuroblastoma cell line; SOX9: SRY-box transcription factor 9; SW620: human Caucasian colon adenocarcinoma; TNF-α: tumour necrosis factor-α; WiDr: derivative of colon adenocarcinoma cell line.

**Table 2:** Summary of reported and predicted biological activities of synthetic fucoxanthin derivatives.

Compound	Structure	Origin	Experimental Model	Observed/Predicted Activity
Isofucoxanthinol (**4**)	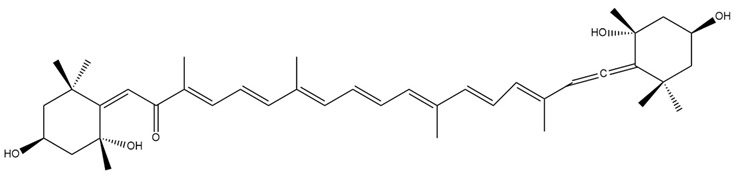	Semi-synthetic	C57BL/6JmsSlc	Observed trend toward reduced body weight and adipose tissue in mice (dietary administration) [139]
Fucoxanthinol hemiketal (**5**)	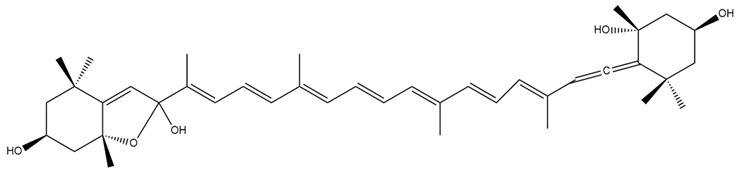	Semi-synthetic	C57BL/6JmsSlc	Observed trend toward reduced body weight and adipose tissue in mice (dietary administration) [139]
Lithocholylfucoxanthin (**6**)	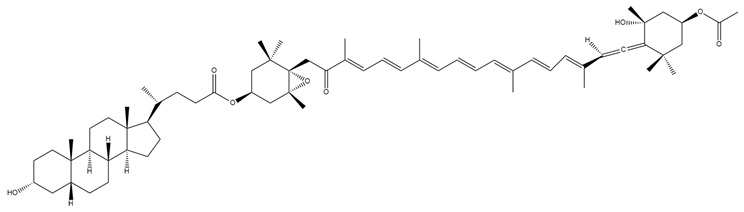	Semi-synthetic	Caco-2 cells	Predicted stronger chemoprotective, anticancer, and antiproliferative activity vs. Fx and FxOH (PASS analysis) [140]
Lithocholyl-fucoxanthinol (**7**)	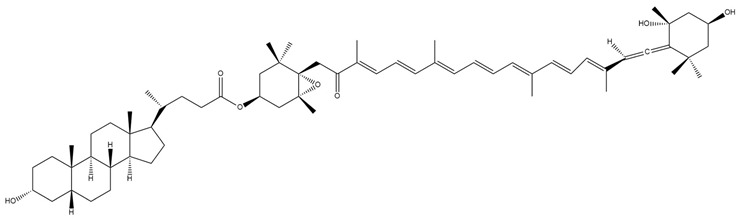	Semi-synthetic	Caco-2 cells	Predicted stronger chemoprotective, anticancer, and antiproliferative activity vs. Fx and FxOH (PASS analysis) [140]
Lithocholylfucoxanthin levulinate (**8**)	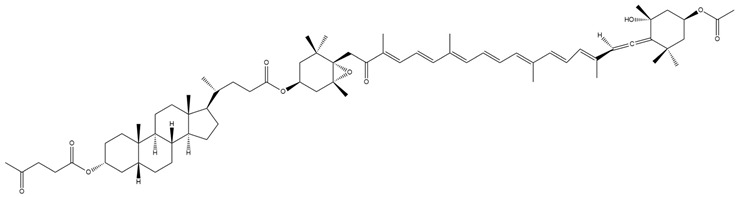	Semi-synthetic	Caco-2 cells	Predicted stronger chemoprotective, anticancer, and antiproliferative activity than Fx and FxOH (PASS analysis) [140]
Lev-lithocholilfucoxanthinol (**9**)	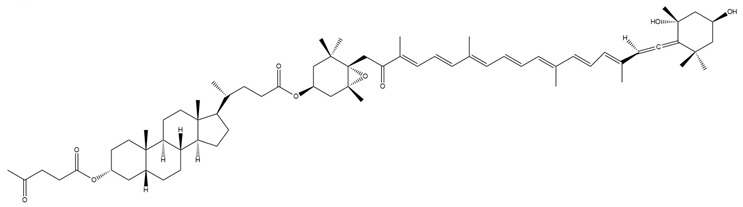	Semi-synthetic	Caco-2 cells	Predicted stronger chemoprotective, anticancer, and antiproliferative activity than Fx and FxOH; similar anti-obesity potential to Fx (PASS analysis) [140]

Legends: C57BL/6JmsSlc: experimental mouse strain; Caco-2: human colorectal adenocarcinoma cells; Fx: fucoxanthin; FxOH: fucoxanthinol; PASS: prediction of activity spectra for substances analysis.

## Data Availability

No new data were created or analyzed in this study. Data sharing is not applicable to this article.

## Abbreviations

The following abbreviations are used in this manuscript:

ARE	Antioxidant response elements
CRTISO	Carotenoid isomerase
CRTISO5	Carotenoid isomerase 5
Fx	Fucoxanthin
FxOH	Fucoxanthinol
GGPP	Geranylgeranyl diphosphate
GGPS	Geranyl pyrophosphate synthase
GST	Glutathione S-transferase
HO-1	Heme oxygenase-1
IPP	Isopentenyl pyrophosphate
IUPAC	International Union of Pure and Applied Chemistry
Keap1	Kelch-like ECH-associated protein 1
LOOH	Lipid hydroperoxide
LPS	Lipopolysaccharide
MEP	Methylerythritol phosphate
MVA	Mevalonate
NAAA	N-acylethanolamine acid amidase
NAFLD	Non-alcoholic fatty liver disease
NF-κB	Nuclear factor kappa B
NIK	NF-κb-inducing kinase
NQO1	NAD(P)H:quinone oxidoreductase-1
Nrf2	Nuclear factor erythroid 2–related factor 2
PDS	Phytoene desaturase
PEA	Palmitoylethanolamide
PPAR-α	Peroxisome proliferator-activated receptor alpha
PPAR-ϒ	Peroxisome proliferator-activated receptor gamma
PSY	Phytoene synthase
ROS	Reactive oxygen species
RXR	9-cis-retinoic acid receptor
SAR	Structure–activity relationship
SOD	Superoxide dismutase
TNF-α	Tumour necrosis factor-α
UCP-1	Uncoupling protein-1
ZDS	Zeaxanthin desaturase
ZEP	Zeaxanthin epoxidase
